# PLLP inhibits the progression of WT p53 gastric cancer by reducing p53 protein ubiquitination by binding to TRIM59

**DOI:** 10.1016/j.jbc.2026.111341

**Published:** 2026-03-04

**Authors:** Zhenhao Quan, Lin Lin, Feipeng Xu, Caijin Zhou, Renwei Huang, Kaiyu Sun, Haiping Jiang

**Affiliations:** 1 Department of General Surgery, The First Affiliated Hospital of Jinan University, Guangzhou, China; 2 Department of Gastrointestinal Surgery, Affiliated Hospital of Guangdong Medical University, Zhanjiang, China

**Keywords:** gastric cancer, plasmolipin, p53, ubiquitination, TRIM59

## Abstract

Gastric cancer (GC) is among the most common malignant tumors worldwide. The inhibition of p53 ubiquitination can inhibit the progression of GC. The mechanism through which plasmolipin (PLLP) regulates p53 ubiquitination in GC remains unclear. In this study, the correlation between PLLP expression and the prognosis of GC was analyzed on the basis of data from The Cancer Genome Atlas database, and the expression characteristics of PLLP and p53 were verified by immunohistochemistry. A PLLP overexpression/knockdown GC cell model was constructed, and cell proliferation, apoptosis, and invasion were detected by Cell Counting Kit-8, flow cytometry, and Transwell assays. Coimmunoprecipitation and Western blotting were used to analyze the PLLP–tripartite motif–containing 59 (TRIM59)–p53 regulatory axis. The antitumor effect of PLLP *in vivo* was verified by tumor formation experiments in nude mice. Cycloheximide tracking assays, coimmunoprecipitation, and ubiquitination analysis were used to determine the effect of PLLP on p53 stability. Combined with bioinformatics prediction and experimental verification, the interaction between PLLP and the E3 ubiquitin ligase TRIM59 and its regulatory effect on the ubiquitination and degradation of p53 were analyzed. Flow cytometry and Transwell assays were used to verify the biological effect of the PLLP–TRIM59–p53 axis. We found that PLLP was downregulated in GC (*p* < 0.05). PLLP interacts with TRIM59, inhibits TRIM59-mediated ubiquitination degradation of p53, and inhibits the progression of GC cells with WT p53. PLLP may be used as a potential biomarker for targeted therapy of GC.

Gastric cancer (GC) is the most common malignant tumor of the digestive system (1). In 2020, there were more than 1 million new cases of GC worldwide, and its incidence and mortality ranked fifth and fourth, respectively, among malignant tumors (2). GC has certain regional characteristics, with a high incidence in East Asia and East Europe. In recent years, the incidence of GC in young patients (aged <50 years) has gradually increased (3). The prognosis of early stage GC is very different from that of advanced-stage GC; because there are no specific symptoms in patients with early stage GC, most of these patients are already in the middle and late stages when diagnosed, making the prevention and treatment of GC difficult (4, 5). Surgical treatment, chemotherapy, and immunotherapy are currently the main comprehensive treatment options for GC. However, tumor metastasis and recurrence often lead to the failure of GC treatment in the middle and late stages (6). Therefore, finding effective and specific molecular markers and drug targets for GC diagnosis to inhibit the progression of this disease is highly important.

The plasmolipin (PLLP) is located on the long arm of chromosome 16 (16q13) and has four exons and a large first intron. The PLLP protein is involved in intracellular transport, lipid raft formation, and tumor metastasis and can activate tumor-related signaling pathways (7, 8). Our previous pan-cancer analysis revealed that PLLP expression was generally increased in gastrointestinal tumors, such as colon adenocarcinoma, esophageal cancer, and rectal adenocarcinoma. However, PLLP expression is decreased in GC, and PLLP may be related to GC progression. In addition, The Cancer Genome Atlas (TCGA) database analysis revealed that the expression of PLLP was positively correlated with the clinical prognosis of patients with GC with WT p53, indicating that there is a connection between PLLP and the p53 signaling pathway, but this connection has not been studied. Although PLLP has been shown to activate the Notch signaling pathway, the crosstalk between Notch signaling and p53 can inhibit tumor cell apoptosis (9). However, the role of PLLP in regulating p53 in GC remains unclear.

The tumor suppressor gene p53 is located on human chromosome 17p13.1, and its encoded protein p53 is a DNA sequence–specific transcription factor. Mutations in the p53 gene are common in human tumors, and 50% of the tissues of patients with GC show positive immunostaining for mutant p53 protein, whereas WT p53 protein is undetectable or very low (10). In tumor cells, WT p53 is often abnormally regulated because of abnormal activity of p53 upstream regulatory genes, epigenetic changes, and post-transcriptional modifications that result in a decrease in p53 protein levels or biological activity, making it unable to function normally as a tumor suppressor gene (11). Among them, the negative regulation of p53 by the E3 ubiquitination ligase MDM2 is the best known; this ubiquitination leads to p53 degradation, thus maintaining low protein levels of p53 and the inactive state of the p53 signaling pathway (12, 13). Therefore, blocking p53 ubiquitination degradation or the interaction with other basic transcription factors to restore the normal function of p53 may inhibit the occurrence or development of GC (14).

Therefore, in this study, we combined *in vitro* and *in vivo* experiments to explore the role and mechanisms through which PLLP inhibits GC progression through the regulation of p53 protein ubiquitination to provide a basis for the diagnosis and treatment of GC.

## Results

### Baseline characteristics of patients with GC

A total of 50 patients were included in this study, with an average age of 61.6 ± 11.6 years. The tumor differentiation was poor, with the majority being poorly differentiated or moderately to poorly differentiated. The Lauren classification was mainly mixed type, followed by diffuse type. Patients with tumors at the local advanced stage (T3/T4) accounted for the majority. A vast majority of patients had lymph node metastasis (N1/N2/N3 stage). The average number of detected lymph nodes was 45.10 ± 18.10, and the average number of lymph node metastases was 11.70 ± 13.70, with a large standard deviation indicating significant individual differences among patients. Many patients (44, 88.0%) had nerve invasion. In addition, 39 patients had Ki67 expression >60%, suggesting that the proportion of actively proliferating tumors in this study cohort was relatively high (Table 1).

**Table 1:** Baseline characteristics of 50 patients with GC

Parameters	Patients
Age (year)	61.6 ± 11.6
Gender	
Men	34 (68.0%)
Women	16 (32.0%)
Tumor size	
>5 cm	32 (64.0%)
≤5 cm	18 (36.0%)
Degree of differentiation	
Low	32 (64.0%)
Low-medium	13 (26.0%)
Medium	4 (8.0%)
Mixed	1 (2.0%)
T staging	
T1	2 (4.0%)
T2	2 (4.0%)
T3	5 (10.0%)
T4	41 (82.0%)
Lymph node metastasis	
N0	6 (12.0%)
N1	8 (16%)
N2	12 (24.0%)
N3	24 (48.0%)
Vascular invasion	
Yes (1)	27 (54.0%)
No (0)	23 (46.0%)
Neural invasion	
Yes (1)	44 (88.0%)
No (0)	6 (12.0%)
Ki67 expression	
>60%	39 (78.0%)
≤60%	11 (22.0%)
Her2 expression	
Positive	15 (30.0%)
Negative	35 (70.0%)
Postoperative survival time (months)	18.70 ± 9.20
Number of lymph nodes	45.10 ± 18.10
Number of lymph node metastases	11.70 ± 13.70
Lauren classification	
Diffuse type	17 (34.0%)
Intestinal type	8 (16.0%)
Mixed type	25 (50.0%)

### Expression and clinical significance of PLLP and p53 in GC

**Figure 1:**
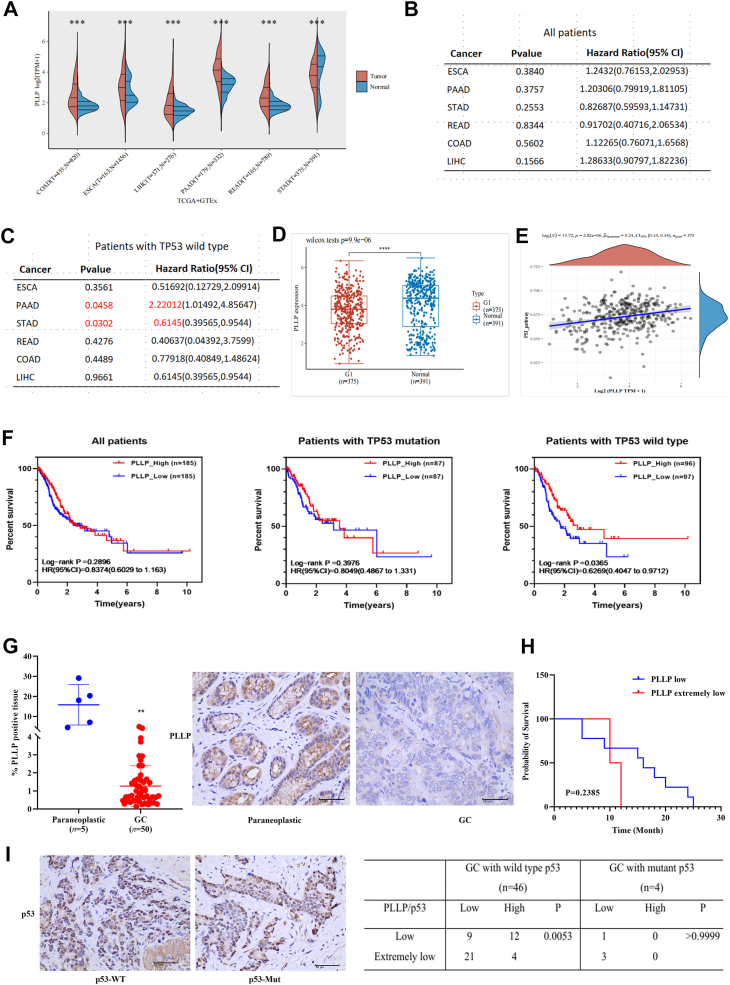
**Expression and clinical significance of PLLP and p53 in GC.***A*–*C*, Pan-cancer analysis. *D*, TCGA database analysis indicated that PLLP was expressed at low levels in GC tissues. *E*, the correlation of PLLP expression with the expression of p53 and its downstream genes in patients with GC in the TCGA database was analyzed. *F*, Kaplan–Meier survival analysis. *G*, percentage of PLLP-positive cells determined by immunohistochemical staining. *H*, survival curves of patients with different levels of PLLP expression. *I*, the percentage of p53-positive cells determined by immunohistochemical staining. ∗∗*p* < 0.01. GC, gastric cancer; PLLP, plasmolipin; TCGA, The Cancer Genome Atlas.

Pan-cancer analysis revealed that the expression of PLLP in gastrointestinal tumors generally increased (colon adenocarcinoma, esophageal cancer, liver hepatocellular carcinoma, pancreatic adenocarcinoma, and rectal adenocarcinoma), whereas in the GC group, the expression of PLLP decreased (Fig. 1*A*). The correlation between PLLP expression and prognosis was positive only for patients with GC and TP53 WT (hazard ratio <1, *p* < 0.05; Fig. 1, *B* and *C*). Analysis of the data from the TCGA database revealed that the expression of PLLP was reduced in GC tissues (Fig. 1*D*) and that the expression of PLLP correlated with the gene transcription of p53 and its downstream signaling pathway (Fig. 1*E*). In addition, Kaplan–Meier survival analysis revealed that the overall survival rate of patients with GC in the high-PLLP expression subgroup did not improve (*p* = 0.2896), and the survival analysis of the p53 mutation subgroup revealed no difference in the overall survival rate of patients with GC in the high-PLLP and low-PLLP expression groups (*p* = 0.3976); however, in the p53 WT subgroups, the overall survival rate of patients with GC in the high-PLLP expression subgroup was greater than in the low-PLLP expression subgroup (*p* = 0.0365, Fig. 1*F*). Immunohistochemical staining revealed that the expression of PLLP was significantly lower in GC tissues than in paracancerous tissues (*p* < 0.01; Fig. 1*G*). On the basis of the average expression levels of PLLP detected by immunohistochemistry, the patients were divided into a PLLP low-expression group and a PLLP extremely low-expression group. The survival curves of the patients, reflecting the changes in survival probability over time (months) at different PLLP expression levels, are shown in Figure 1*H*. The statistical test results revealed that the *p* value was 0.2385, but the survival time of patients in the low-expression group was still longer than that in the extremely low-expression group. Expression of p53 WT was higher than that of the p53 mutant type (Fig. 1*I*), suggesting that PLLP may affect p53 WT in GC.

### PLLP expression in GC cells with WT p53 and mutant p53 and its effect on GC cell proliferation and invasion

**Figure 2:**
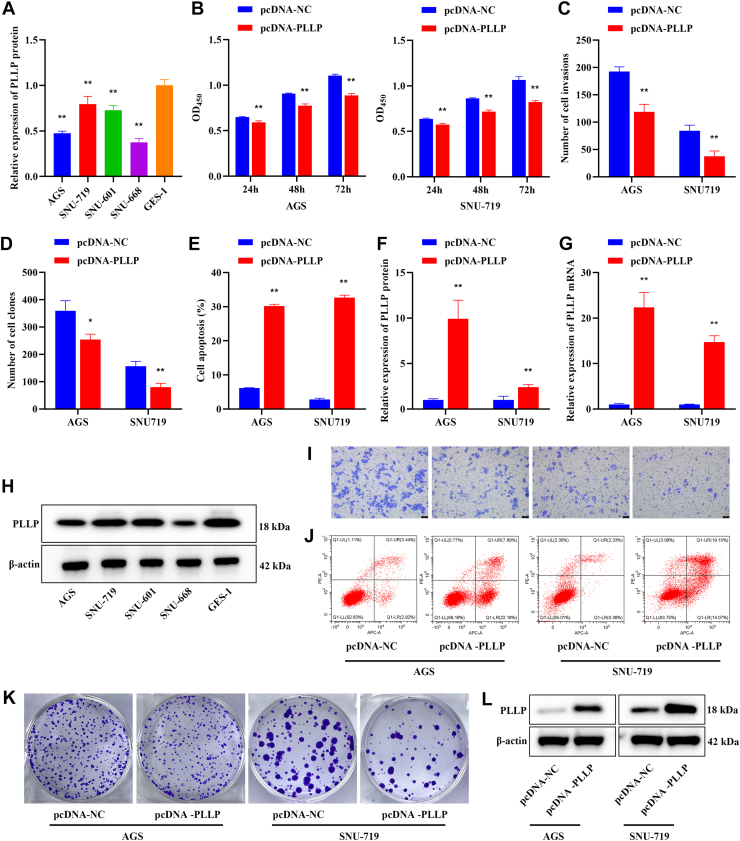
**PLLP expression in GC cells with WT p53 and mutant p53 and its effect on GC cell proliferation and invasion.***A*, PLLP expression in different GC cell lines (*n* = 3). *B*, CCK-8 was used to assess cell activity at 24, 48, and 72 h (*n* = 3). *C*, Transwell assay to detect cell invasive ability (*n* = 3). *D*, a cell cloning assay was used to detect cell proliferation ability (*n* = 3). *E*, detection of apoptosis by flow cytometry (*n* = 3). *F*, expression of PLLP detected by Western blot (*n* = 3). *G*, expression of PLLP measured by qRT–PCR (*n* = 3). *H*, PLLP-expressing protein bands. *I*, representative images of the Transwell experiment (crystal violet staining). *J*, representative flow cytometry images of apoptosis. *K*, representative images of the cell cloning assay. *L*, PLLP-expressing protein bands. Compared with the GES-1 or pcDNA-NC group, ∗*p* < 0.05, ∗∗*p* < 0.01. CCK-8, Cell Counting Kit-8; NC, negative control; PLLP, plasmolipin; qRT–PCR, quantitative RT–PCR.

First, TP53 mutation and PLLP expression were examined in various GC cell lines, revealing that TP53 was mutated in SNU-601 and SNU-668 cells (Table 2). According to the results of the Western blot analysis, PLLP expression was significantly lower in the GC cell lines than in the GES-1 cells (*p* < 0.01, Fig. 2, *A* and *H*). The effects of stable overexpression of PLLP on the growth, invasion, and programmed cell death of TP53 WT GC cells were examined. Compared with the pcDNA-negative control (NC) group, the pcDNA-PLLP group exhibited significantly reduced cell activity (*p* < 0.01, Fig. 2*B*), inhibited cell invasion (*p* < 0.01, Fig. 2, *C* and *I*), reduced cell proliferation (*p* < 0.05, Fig. 2, *D* and *K*), and increased apoptosis (*p* < 0.01, Fig. 2, *E* and *J*). Moreover, quantitative RT–PCR (qRT–PCR) and Western blot analyses revealed that stable overexpression of PLLP significantly increased the level of PLLP (*p* < 0.01, Fig. 2, *F*, *G* and *L*), demonstrating that stable overexpression of PLLP significantly inhibited the proliferation and invasion of TP53 WT GC cells.

**Table 2:** TP53 mutation status

Cell line	TP53 gene mutation
AGS	WT
SNU-719	WT
SNU-601	p.Arg273His (c.818G>A)
SNU-668	p.Ser215Asn (c.644G>A)

**Figure 3:**
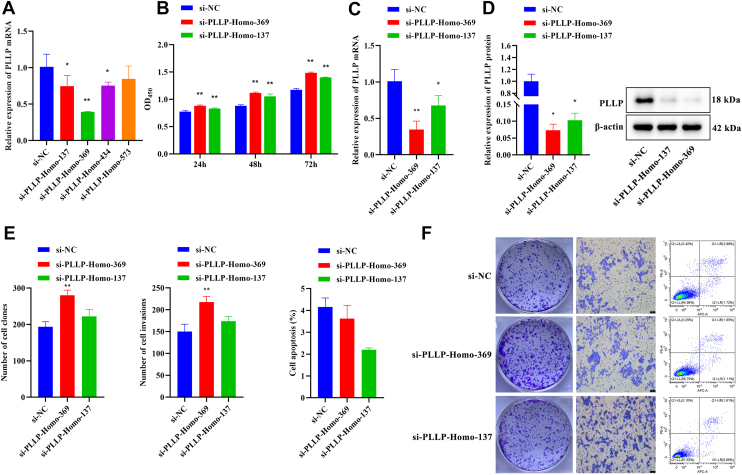
**Effects of PLLP knockdown on GES-1 cell proliferation, invasion, and apoptosis.***A*, expression of PLLP measured by qRT–PCR (*n* = 3). *B*, CCK-8 was used to assess cell activity at 24, 48, and 72 h (*n* = 4). *C*, expression of PLLP measured by qRT–PCR (*n* = 3). *D*, expression of PLLP detected by Western blot (*n* = 3). *E*, cell proliferation, invasion, and apoptosis capabilities were assessed using cell cloning assays, Transwell assays, and flow cytometry (*n* = 3). *F*, representative images from cell cloning experiments, Transwell assays (crystal violet staining), and apoptosis assays. Compared with the si-NC group, ∗*p* < 0.05, ∗∗*p* < 0.01. CCK-8, Cell Counting Kit-8; NC, negative control; PLLP, plasmolipin; qRT–PCR, quantitative RT–PCR.

In addition, we performed PLLP knockdown experiments in GES-1 cells. Based on qRT–PCR analysis, the si-PLLP-Homo-369 and si-PLLP-Homo-137 sequences demonstrated superior knockdown efficiency and were subsequently used in all subsequent experiments (*p* < 0.01, Fig. 3*A*). Compared with the si-NC control group, PLLP knockdown significantly increased cell viability (*p* < 0.01, Fig. 3*B*). qRT–PCR and Western blot analyses demonstrated that PLLP knockdown significantly reduced PLLP expression (*p* < 0.01, Fig. 3, *C* and *D*). Furthermore, compared with the si-NC control group, si-PLP-Homo-369 significantly enhanced cell proliferation and invasion capabilities while having no significant effect on apoptosis (*p* < 0.01, Fig. 3, *E* and *F*). This confirms that PLLP knockdown promotes the activity and proliferation of GES-1 cells.

### WT p53 mediates the effects of PLLP on cell proliferation and invasion in GC cells

**Figure 4:**
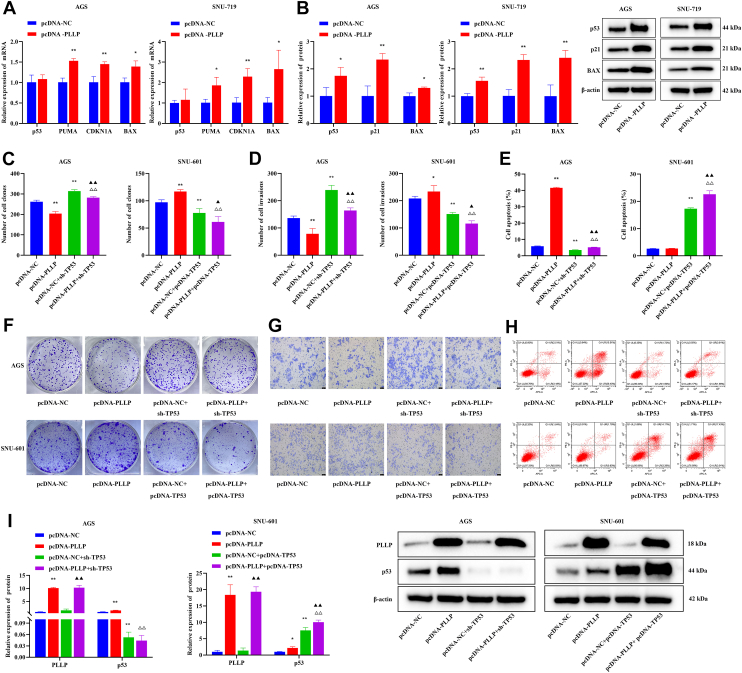
**WT p53 mediates the effects of PLLP on cell proliferation and invasion in GC cells.***A*, expression of p53, PUMA, CDKN1A, and BAX measured by qRT–PCR in AGS and SNU-719 cells (*n* = 3). *B*, expression of p53, p21, and BAX detected by Western blot in AGS and SNU-719 cells (*n* = 3). *C*, a cell cloning assay was used to detect cell proliferation ability (*n* = 3). *D*, Transwell assay to detect cell invasive ability (*n* = 3). *E*, detection of apoptosis by flow cytometry (*n* = 3). *F*, representative images of the cell cloning assay (*n* = 3). *G*, representative images of the Transwell experiment (crystal violet staining). *H*, representative flow cytometry images. *I*, expression of PLLP and p53 detected by Western blot in AGS and SNU-601 cells. Compared with the pcDNA-NC group, ∗*p* < 0.05, ∗∗*p* < 0.01; compared with the pcDNA-PLLP group, ^△△^*p* < 0.01; and compared with the pcDNA-NC + sh-TP53 group, ^▲^*p* < 0.05, ^▲▲^*p* < 0.01. BAX, Bcl-2-associated X protein; GC, gastric cancer; PLLP, plasmolipin; qRT–PCR, quantitative RT–PCR.

Analysis of the transcriptional levels of p53 and its related genes in GC cells with stable overexpression of PLLP revealed that compared with the pcDNA-NC group, the pcDNA-PLLP group had significantly elevated mRNA expression of PUMA, CDKN1A, and Bcl-2-associated X protein (BAX) (*p* < 0.05, Fig. 4*A*) and significantly increased protein expression of p53, p21, and BAX (*p* < 0.05, Fig. 4*B*). Furthermore, compared with the pcDNA-PLLP group, the pcDNA-PLLP+ sh-TP53 group had significantly increased proliferation (*p* < 0.01, Fig. 4, *C* and *F*), invasion (*p* < 0.01, Fig. 4, *D* and *G*), and inhibited apoptosis (*p* < 0.01, Fig. 4, *E* and *H*). However, the proliferation (*p* < 0.01, Fig. 4, *C* and *F*), invasion (*p* < 0.01, Fig. 4, *D* and *G*), and apoptosis (*p* < 0.01, Fig. 4, *E* and *H*) were significantly inhibited in the pcDNA-PLLP+ pcDNA-TP53 group. Furthermore, compared with the pcDNA-PLLP group, the pcDNA-PLLP+ sh-TP53 group had significantly decreased expression of p53 in AGS cells, and the pcDNA-PLLP+ pcDNA-TP53 group had significantly increased expression of p53 in SNU-601 cells (*p* < 0.01, Fig. 4*I*), suggesting that p53 plays a tumor suppressor role of PLLP in TP53 WT GC cells.

### WT p53 mediates the effect of PLLP in GC cells on tumor proliferation in GC mice

**Figure 5:**
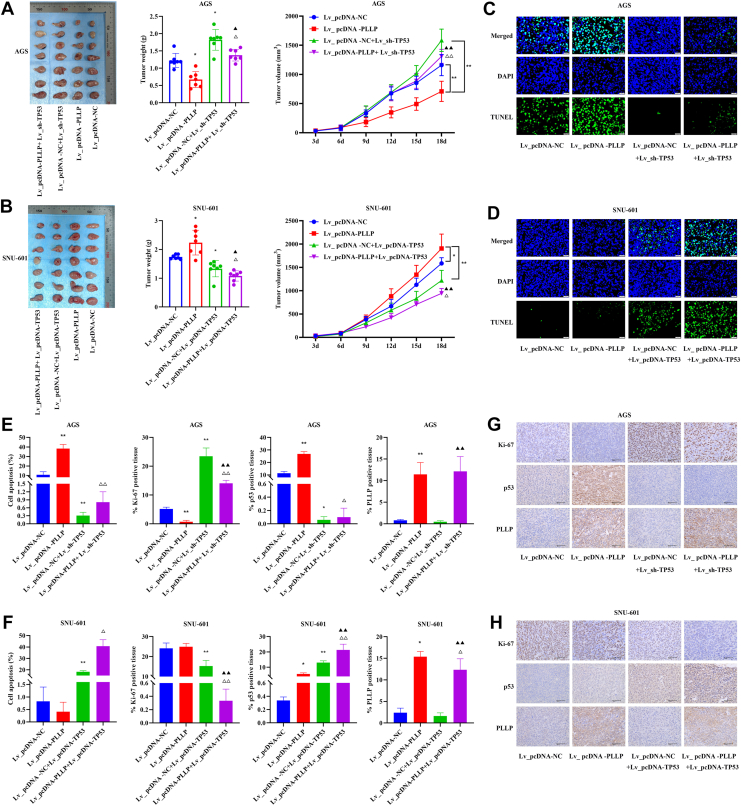
**WT p53 mediates the effect of PLLP in GC cells on tumor proliferation in mice with GC.***A*, tumor image, tumor weight, and tumor volume in AGS cell tumor–bearing mice (*n* = 7). *B*, tumor image, tumor weight, and tumor volume in SNU-601 cell tumor–bearing mice (*n* = 7). *C*, TUNEL staining of tumor tissue from AGS cell tumorigenic mice (400×; 20 μm; *n* = 3). *D*, TUNEL staining of tumor tissue from SNU-601 cell tumorigenic mice (400×; 20 μm; *n* = 3). *E*, apoptosis rate and Ki-67, PLLP, and p53 expression in AGS cell tumor–bearing mice (*n* = 3). *F*, apoptosis rate and Ki-67, PLLP, and p53 expression in SNU-601 cell tumor–bearing mice (*n* = 3). *G*, immunohistochemical staining of Ki-67, PLLP, and p53 in tumor tissues from AGS cell tumor–bearing mice (40×; 50 μm). *H*, immunohistochemical staining of Ki-67, PLLP, and p53 in tumor tissues from SNU-601 tumor–bearing mice (40×; 50 μm). Compared with the pcDNA-NC group, ∗*p* < 0.05, ∗∗*p* < 0.01; compared with the pcDNA-PLLP group, ^△△^*p* < 0.01; compared with the pcDNA-NC + sh-TP53 group, ^▲^*p* < 0.05, ^▲▲^*p* < 0.01. GC, gastric cancer; NC, negative control; PLLP, plasmolipin.

In this study, we performed *in vivo* experimental analyses, and the results revealed that, compared with the Lv_pcDNA-NC group, the Lv_pcDNA-PLLP group had significantly lower tumor weight and tumor volume in AGS cell tumorigenic mice but greater tumor weight and tumor volume in SNU-601 cell tumorigenic mice (*p* < 0.05; Fig. 5, *A* and *B*). Compared with those in the Lv_pcDNA-PLLP group, the tumor weights and tumor volumes of the mice in the Lv_pcDNA-PLLP+ Lv_sh-TP53 group were significantly greater, and those of the mice in the Lv_pcDNA-PLLP+ Lv_pcDNA-TP53 group were significantly lower (*p* < 0.05; Fig. 5, *A* and *B*). In addition, compared with the Lv_pcDNA-NC group, the Lv_ pcDNA-PLLP group significantly promoted apoptosis and decreased the expression of Ki-67 in tumor tissues from AGS cell tumor–bearing mice. Compared with the Lv_pcDNA-PLLP group, Lv_pcDNA-PLLP+ Lv_sh-TP53 inhibited the antitumor effect of Lv_pcDNA-PLLP (*p* < 0.05; Fig. 5, *C* and *H*), indicating that WT p53 mediates the antitumor effects of PLLP.

### Regulation of the ubiquitination of WT p53 in GC by PLLP

**Figure 6:**
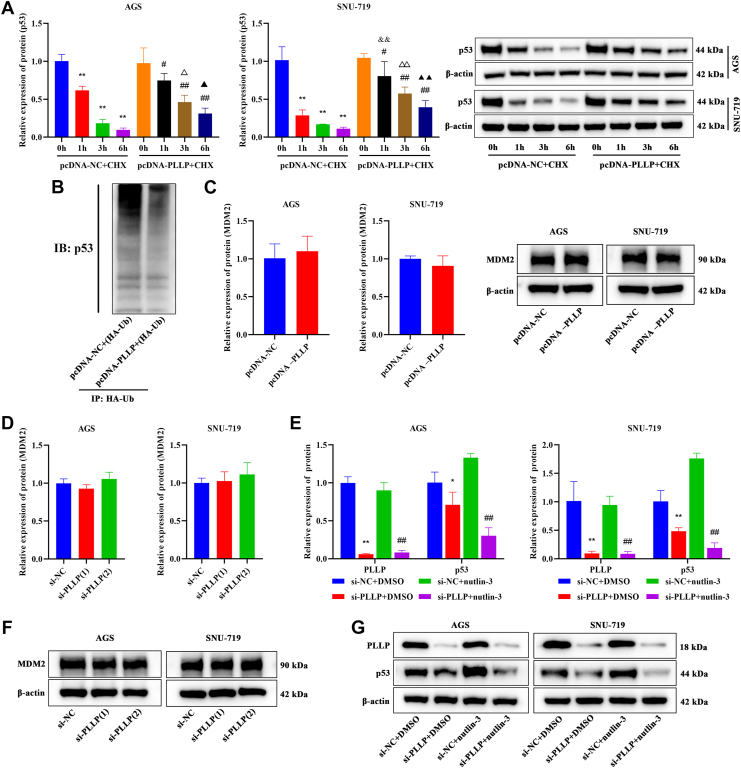
**Regulation of the ubiquitination of WT p53 in GC by PLLP.***A*, p53 protein degradation in the cells was detected by Western blotting (*n* = 3). *B*, Co-IP was used to detect the ubiquitination of the p53 protein. *C*, Western blotting was used to detect the protein level of MDM2 after PLLP overexpression (*n* = 3). *D*, Western blotting was used to detect the protein level of MDM2 after PLLP knockdown (*n* = 3). *E*, the protein levels of PLLP and p53 were detected by Western blotting (*n* = 3). *F* and *G*, protein band. Compared with the pcDNA-NC + CHX (0 h) or si-NC + DMSO group, ∗∗*p* < 0.01; compared with the pcDNA-PLLP + CHX (0 h) or si-NC + nutlin-3 group, #*p* < 0.05, ##*p* < 0.01; compared with the pcDNA-NC + CHX (1 h), &&*p* < 0.01; compared with the pcDNA-NC + CHX (3 h), ^△^*p* < 0.05, ^△△^*p* < 0.01; and compared with the pcDNA-NC + CHX (6 h), ^▲^*p* < 0.05, ^▲▲^*p* < 0.01. Co-IP, coimmunoprecipitation; DMSO, dimethyl sulfoxide; GC, gastric cancer; PLLP, plasmolipin.

When the effect of PLLP expression on p53 protein degradation in GC cells was evaluated, a significant increase in p53 protein expression was detected in the pcDNA-PLLP + cycloheximide (CHX) group compared with the pcDNA-NC + CHX group (*p* < 0.05, Fig. 6*A*). The results of p53 protein ubiquitination are shown in Figure 6*B*. p53 protein binding to hemagglutinin (HA)-ubiquitin (Ub) extracted proteins was detected, and p53 protein expression was greater in the pcDNA-NC group than in the pcDNA-PLLP group. The effects of stable overexpression of PLLP or transient knockdown of PLLP by siRNA on the expression of MDM2, a negative feedback regulator of p53, were not significant (*p* > 0.05, Fig. 6, *C*, *D* and *F*). We subsequently revealed that in the presence of nutlin-3, knockdown of PLLP still significantly reduced the level of p53 (*p* < 0.01, Fig. 6, *E* and *G*), indicating that the regulation of p53 by PLLP is not dependent on the MDM2 pathway. PLLP may act as a new p53 stabilizing factor, inhibiting the degradation of p53 through a nonclassical ubiquitination pathway, thereby enhancing its tumor suppressor function.

### PLLP regulates the ubiquitination and degradation of the p53 protein by binding to tripartite motif–containing 59, thereby mediating the occurrence and development of tumors

**Figure 7:**
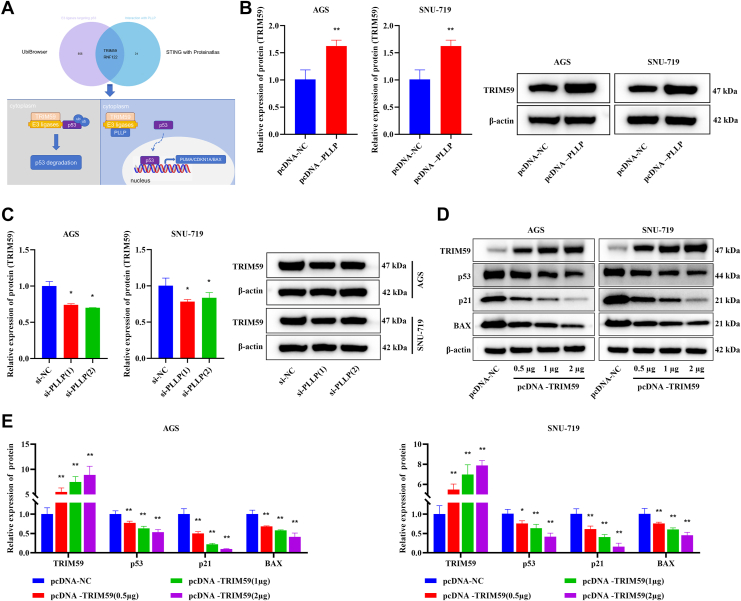
**PLLP regulates the ubiquitination and degradation of the p53 protein by binding to TRIM59, thereby mediating the occurrence and development of tumors.***A*, datasets were used to analyze the relationship between PLLP and p53 ubiquitination degradation (*n* = 3). *B*, Western blotting was used to analyze the effect of PLLP overexpression on TRIM59 protein expression (*n* = 3). *C*, Western blotting was used to analyze the effect of PLLP knockdown on TRIM59 protein expression (*n* = 3). *D* and *E*, Western blotting was used to analyze the effect of TRIM59 overexpression on the p53 signaling pathway (*n* = 3). Compared with the pcDNA-NC or si-NC group, ∗*p* < 0.05, ∗∗*p* < 0.01. NC, negative control; PLLP, palsmolipin; TRIM59, tripartite motif–containing 59.

UbiBrowser predicted a total of 555 E3 Ub ligases targeting p53, and STING with ProteinAtlas predicted a total of 33 PLLP-binding proteins, including 2 E3 Ub ligases that cobind: tripartite motif–containing 59 (TRIM59) and RNF122. TRIM59 can bind to p53 and promote its ubiquitination and degradation (Fig. 7*A*). Compared with the pcDNA-NC group, the pcDNA-PLLP group presented significantly increased expression of the TRIM59 protein (*p* < 0.01) (Fig. 7*B*). In contrast, compared with the si-NC group, the si-PLLP (1) and si-PLLP (2) groups presented significant decreases in TRIM59 protein expression (*p* < 0.05, Fig. 7*C*). These results consistently indicate that PLLP is able to positively regulate TRIM59 protein expression. In addition, the effect of TRIM59 overexpression on the p53 signaling pathway was observed. Compared with those in the pcDNA-NC group, the expression of TRIM59 was significantly greater, and the levels of p53, p21, and BAX were significantly lower in the pcDNA-TRIM59 (0.5 μg), pcDNA-TRIM59 (1 μg), and pcDNA-TRIM59 (2 μg) groups (*p* < 0.05, Fig. 7, *D* and *E*), suggesting that TRIM59 may play a biological role by downregulating the expression of p53 and its downstream target genes p21 and BAX.

**Figure 8:**
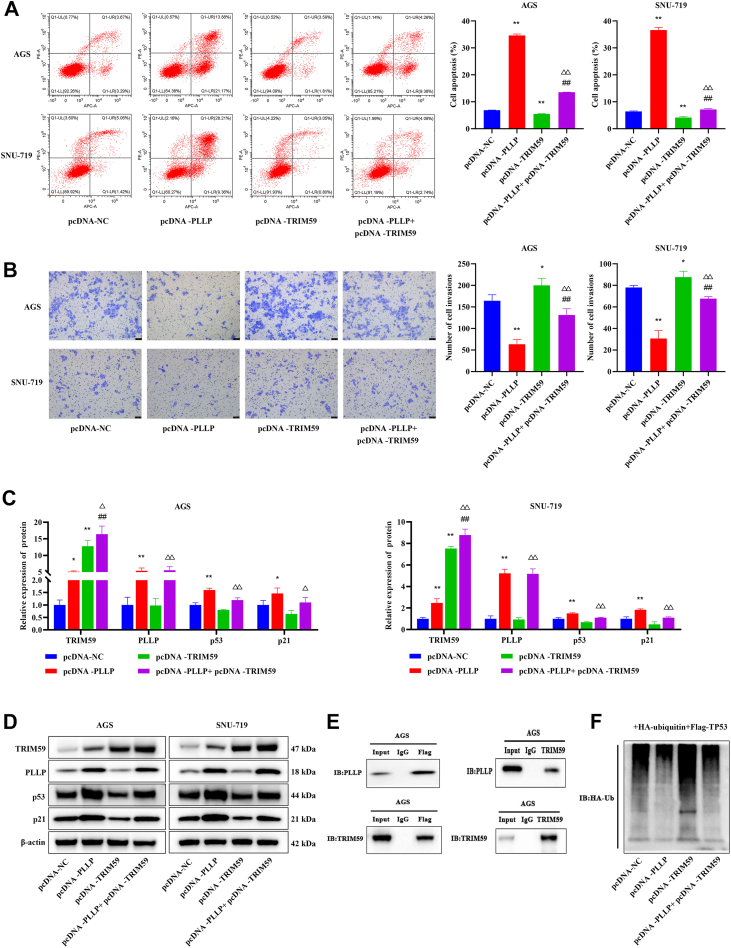
**PLLP regulates the ubiquitination and degradation of the p53 protein by binding to TRIM59, thereby mediating the occurrence and development of tumors.***A*, apoptosis was detected by flow cytometry (*n* = 3). *B*, Transwell assays were used to detect cell invasion (crystal violet staining, *n* = 3). *C* and *D*, Western blotting was used to detect the protein expression of TRIM59, PLLP, p53, and p21 (*n* = 3). *E*, protein binding of PLLP to TRIM59 was analyzed by Co-IP. *F*, Co-IP was used to detect p53 protein ubiquitination. Compared with the pcDNA-NC group, ∗*p* < 0.05, ∗∗*p* < 0.01; compared with the pcDNA-PLLP group, ##*p* < 0.01; compared with the pcDNA-TRIM59 group, ^△^*p* < 0.05, ^△△^*p* < 0.01. Co-IP, coimmunoprecipitation; NC, negative control; PLLP, palsmolipin; TRIM59, tripartite motif–containing 59.

In addition, the effect of PLLP on tumor proliferation and invasion was observed through the regulation of p53 deubiquitination through TRIM59. Compared with that in the pcDNA-PLLP group, apoptosis was significantly lower (*p* < 0.01, Fig. 8*A*), and cell invasion was significantly greater (*p* < 0.01, Fig. 8*B*) in the pcDNA-PLLP + pcDNA-TRIM59 group. Moreover, PLLP overexpression significantly upregulated the expression of p53 and its downstream target gene p21 (*p* < 0.05), whereas TRIM59 had the opposite regulatory effect (Fig. 8, *C* and *D*). The results in Figure 8*E* show that the TRIM59 protein bound to the enriched PLLP protein. In further experiments, the FLAG-p53 protein in each group was bound to HA-Ub and ubiquitinated. Among them, the HA-Ub signal of the pc-DNA + PLLP+ (HA-Ub + FLAG-p53) group was the weakest, suggesting that PLLP may inhibit p53 ubiquitination (Fig. 8*F*). Together, these findings suggest that the TRIM59 protein interacts with the PLLP protein and that PLLP reduces p53 ubiquitination by binding to TRIM59, which in turn affects GC cell proliferation and invasion.

## Discussion

In this study, we revealed that PLLP expression was low in GC and was positively correlated with the prognosis of patients with WT p53. Overexpression of PLLP inhibited the proliferation and invasion of GC cells, and WT p53 mediated the tumor-suppressive effect of PLLP on GC. PLLP enhances the stability of p53 by reducing its ubiquitination level, but its regulation of p53 is independent of the MDM2 pathway. Moreover, PLLP positively regulated the expression level of the TRIM59 protein, and PLLP antagonized its ubiquitination of p53 by binding to TRIM59. The PLLP–TRIM59–p53 axis affects the apoptosis process of WT p53 GC cells.

PLLP is a membrane protein, and its mechanism of action in GC remains unclear. As a negative regulator of the cell cycle, p53 participates in the regulation of key biological processes, such as cell cycle progression, DNA damage repair, cell differentiation, and apoptosis (15). WT p53 has a transactivation function and can widely inhibit tumorigenesis, but its stability is poor, and its half-life is only a few minutes (16). In human cancers, mutation or loss of the p53 gene occurs frequently and is strongly associated with tumor development and growth (17). Clinical studies have shown that abnormal overexpression of p53 protein indicates that tumors have a high risk of metastasis, recurrence, and poor prognosis (18). In this study, PLLP expression was low in GC tissues, and its high expression was associated with the survival rate of patients with WT p53; moreover, overexpression of PLLP significantly promoted apoptosis and inhibited proliferation and invasion in AGS GC cells with WT p53. These findings are consistent with the central role of p53 in maintaining genome stability and regulating apoptosis reported in previous studies (19). Notably, p53 knockdown did not affect the expression level of PLLP, indicating that PLLP is upstream of p53 signaling. In addition, in p53-mutant GC cells (SNU-601), the coexpression of PLLP and WT p53 produced a synergistic tumor-suppressor effect, suggesting that the regulation of p53 by PLLP may be important for maintaining cellular homeostasis.

Loss of p53 function is considered to be the main cause of uncontrolled tumor cell proliferation (20). In the present study, PLLP significantly delayed the degradation of the p53 protein, and this effect was more intense in SNU-719 cells. In addition, the finding that overexpression of PLLP reduces the ubiquitination level of p53 provides important clues to explain the mechanism by which PLLP stabilizes p53. MDM2, an E3 Ub ligase that increases p53 protein ubiquitination and degradation, has attracted attention as a strategy to induce p53 protein accumulation in patients with WT p53 tumors (21). Overexpression of MDM2 is a major factor leading to p53 protein degradation in patients with WT p53 (22). In addition, MDM2 can also regulate p53 function in a nonubiquitination-dependent manner, such as through direct inhibition of p53 transcriptional activity (23). Since p53 triggers apoptosis when DNA is irremediated, inducing p53 protein accumulation has become an attractive antitumor treatment strategy, which also promotes small-molecule inhibitors targeting the MDM2–p53 interaction to become a research hotspot in tumor therapy (24, 25). The nutlin family is a nongenotoxic small molecule–specific inhibitor of MDM2–p53 binding, which promotes the accumulation of p53 protein and mediates antitumor effects (26). In this study, PLLP overexpression did not affect MDM2 levels, and interestingly, although PLLP did not affect MDM2 expression, the combination of nutlin-3 and PLLP knockdown still significantly reduced cellular p53 levels, suggesting that PLLP may regulate p53 *via* other E3 Ub ligases or deubiquitinating enzymes. Future studies may incorporate mass spectrometry to identify the proteins that interact with PLLP.

TRIM59 is an important member of the TRIM protein family, which is characterized by E3 Ub ligase activity and plays a key role in protein homeostasis regulation (27). TRIM59 is overexpressed in a variety of malignant tumors (including GC, lung cancer, and breast cancer) and is closely related to poor prognosis (28, 29). We found that PLLP interacted with TRIM59 and that overexpression of PLLP increased the level of TRIM59, but PLLP and TRIM59 had antagonistic effects on function. In addition, PLLP can significantly inhibit TRIM59-mediated p53 ubiquitination, which is consistent with the previously reported function of TRIM59 as a promoter of p53 ubiquitination (10). These findings suggest that PLLP may inhibit its E3 ligase activity by binding to TRIM59 and reducing the ubiquitination-mediated degradation of p53. This seemingly contradictory phenomenon suggests that PLLP may have a complex regulatory effect on TRIM59. We speculate that the interaction between PLLP and TRIM59 may, on the one hand, increase the stability of TRIM59 and, on the other hand, inhibit its E3 Ub ligase activity through steric hindrance or allosteric effects. This needs to be further clarified in future research. CDKN1A is a key downstream effector of the p53 signaling pathway, and its encoded p21 protein is an important cyclin-dependent kinase inhibitor. Studies have reported that the expression level of p21 is closely related to the differentiation, proliferation, and metastatic potential of tumor cells. p53 initiates the apoptotic program mainly by activating the proapoptotic protein (BAX) (24). In this study, overexpression of PLLP upregulated the expression of the p53 downstream genes, BAX, p21, and PUMA. Furthermore, overexpression of TRIM59 downregulated the expression of p53 downstream genes (BAX and p21), suggesting that TRIM59 may also have a transcriptional regulatory mechanism independent of ubiquitination. These findings echo recent reports that E3 ligases have "enzyme activity–independent functions" (30) and provide a new perspective for understanding the versatility of TRIM59.

**Figure 9:**
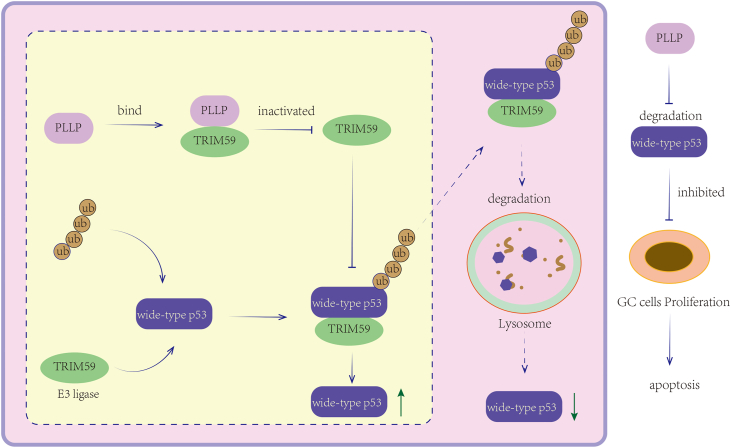
**Mechanistic diagram of this study**.

In conclusion, we report that PLLP is expressed at low levels in GC cells, that PLLP overexpression can significantly inhibit the malignant phenotype of GC cells, and that PLLP inhibits E3 ligase activity by binding to TRIM59, reducing the ubiquitination-mediated degradation of p53, and affects the apoptotic process of GC cells with WT p53. The PLLP expression level may be used as a potential biomarker to predict the response of patients with GC to p53-targeted therapy. The mechanism diagram for this study is shown in Figure 9. However, the following aspects still need to be further explored: More evidence on how PLLP regulates TRIM59 function is lacking, and whether PLLP regulates other E3 ligases is unknown and has not been further verified through PLLP knockout experiments. Furthermore, this study lacks validation of patient survival, and the xenograft duration was relatively short. Future studies will contribute to a more complete understanding of the complexity of this regulatory network.

## Experimental procedures

### TCGA dataset

The Reads Per Kilobase of transcript per Million mapped reads values of PLLP in the TCGA database (https://tcga-data.nci.nih.gov/tcga/) were downloaded. There were 375 GC tissues and 391 normal tissues. The expression of PLLP in gastrointestinal tumors was analyzed. The relationship between the PLLP expression level and the prognosis of patients with GC in the TCGA database was analyzed, and the relationship between the PLLP expression level and the outcome of GC patients with WT p53 was analyzed.

### Cancer Cell Line Encyclopedia and Cellosaurus Databases

The Cancer Cell Line Encyclopedia Database (https://portals.broadinstitute.org/ccle/about) is an online encyclopedia of a compilation of gene expression, chromosomal copy number, and massively parallel sequencing data from 947 human cancer cell lines (31). According to the Cancer Cell Line Encyclopedia and Cellosaurus (https://www.cellosaurus.org) Database analysis, p53 mutations in GC cell lines were identified.

### UbiBrowser and STING dataset

The UbiBrowser database was used to screen possible E3 ligases related to p53 (https://ubibrowser.ncpsb.org/). The STRING database (https://string-db.org/) was used to identify PLLP-interacting proteins.

### Tissue sample collection

A total of 50 patients who underwent GC tissue resection at the Department of Gastrointestinal Surgery, Affiliated Hospital of Guangdong Medical University, from January 2020 to June 2023 were included. GC tissues from the same patient were selected as the experimental group, and adjacent tissues (2 cm from the edge of the tumor) were selected as the control group. All the specimens were embedded in paraffin and cut into 3 μm sections for immunohistochemical staining. The procedures followed were in accordance with the approval of the Medical Ethics Committee of the Affiliated Hospital of Guangdong Medical University (KT2023-073-02).

### Cell experiments

Human GC cell lines (AGS, SNU-719, SNU-601, and SNU-668) and human normal gastric mucosal epithelial cells (GES-1) were obtained from Shanghai iCell, which provides short tandem repeat–verified stocks. No further authentication was performed in-house. The cells were cultured in RPMI1640 medium (Wuhan Procell) supplemented with 10% fetal bovine serum (Shanghai Vivacell) and 1% penicillin‒streptomycin (Shanghai BasalMedia) at 37 °C and 5% CO_2_. Cells were divided into the following groups: (i) pcDNA-NC, pcDNA-PLLP; (ii) pcDNA-NC, pcDNA-PLLP, pcDNA-NC + sh-TP53, and pcDNA-PLLP + sh-TP53; (iii) pcDNA-NC, pcDNA-PLLP, pcDNA-NC + pcDNA-TP53, and pcDNA-PLLP + pcDNA-TP53; (iv) pcDNA-NC + CHX (50 μg/ml CHX for 0, 1, 3, and 6 h), pcDNA-PLLP + CHX (50 μg/ml CHX for 0, 1, 3, and 6 h); (v) si-NC (sequences 5′-UUCUCCGAACGUGUCACGUTT-3′, 5′-ACGUGACACGUUCGGAGAATT-3′), si-PLLP-Homo-137 (1) (sequences 5′-GAGUUCCCGUCGAAAGUUATT-3′, 5′-UAACUUUCGACGGGAACUCTT-3′), si-PLLP-Homo-369 (2) (sequences 5′-CAAUCGUCCUCUUCAACCUTT-3′, 5′-AGGUUGAAGAGGACGAUUGTT-3′); (vi) si-NC + dimethyl sulfoxide (Bomei); si-PLLP + dimethyl sulfoxide; si-NC + nutlin-3 (an inhibitor of MDM2 and p53 interaction, Sigma‒Aldrich); si-PLLP + nutlin-3; (vii) pcDNA-NC, pcDNA-TRIM59 (0.5 μg), pcDNA-TRIM59 (1 μg), and pcDNA-TRIM59 (2 μg); and (viii) pcDNA-NC, pcDNA-PLLP, pcDNA-TRIM59, and pcDNA-PLLP + pcDNA-TRIM59. GES-1 cells were divided into the following groups: (i) si-NC (sequences 5′-UUCUCCGAACGUGUCACGUTT-3′, 5′-ACGUGACACGUUCGGAGAATT-3′), si-PLLP-Homo-137 (sequences 5′-GAGUUCCCGUCGAAAGUUATT-3′, 5′-UAACUUUCGACGGGAACUCTT-3′), si-PLLP-Homo-369 (sequences 5′-CAAUCGUCCUCUUCAACCUTT-3′, 5′-AGGUUGAAGAGGACGAUUGTT-3′), si-PLLP-Homo-434 (sequences 5′-CACUGGUGUUAAUGAUCUUTT-3′, 5′-AAGAUCAUUAACACCAGUGTT-3′), and si-PLLP-Homo-573 (sequences 5′-GUUUGGUGAUGAUCGCCUATT-3′, 5′-UAGGCGAU CAUCACCAAACTT-3′. (ii) si-NC, si-PLLP-Homo-369, and si-PLLP-Homo-137. Lentiviral packaging was used to overexpress PLLP, TP53, or TRIM59 expression vectors or shRNA or siRNA to knock down PLLP or TP53, completed by Shanghai GENE, and to transfect empty plasmids (pcDNA-NC or si-NC) as NCs. Cell transfections were performed using Lipofectamine 3000 transfection reagent (Thermo Fisher Scientific). In short, the cell density was adjusted to 2 × 10^5^ cells/ml, and 2 ml/well was inoculated into 6-well plates for incubation at 37 °C under 5% CO_2_ conditions. Next, 75 pmol of siRNA was diluted with 17 μl of buffer, 7.5 μl of plus transfection reagent was added, and the mixture was mixed well to obtain the siRNA/plus complex. After the cells adhered to the well, the supernatant in the wells was removed, and the aforementioned transfection complex was added to the culture medium and mixed well before it was added to the well plate. The cells were cultivated at 37 °C and 5% CO_2_ in a constant-temperature incubator. In addition, for viral infection, the cell density was adjusted to 3 × 10^4^ cells/ml, and 2 ml/well was inoculated into 6-well plates. The plates were cultured at 37 °C under 5% CO_2_ conditions. The infection efficiency was approximately 80%, and the corresponding infection conditions (judged under the microscope) and multiplicity of infection served as the basis for subsequent infection experiments. On the basis of the cell multiplicity of infection (SNU-601: 50; SNU-719: 50; and AGS: 20) and virus titer (5 × 10^8^ TU/ml), the virus volume was increased (8 μl). The cells were cultivated at 37 °C and 5% CO_2_ in a constant-temperature incubator. After 16 h of infection, the culture medium was replaced, and the cells were cultivated at 37 °C and 5% CO_2_ for 72 h.

### Mouse GC transplantation tumor model

SPF BALB/c-nude mice (males, 4 weeks old, n = 56) were provided by Chengdu GemPharmatech. The feeding conditions included a temperature ranging from 20 °C to 26 °C, relative humidity ranging from 30% to 70%, and a 12 h day/12 h night light cycle. After 3 days of adaptive feeding, the experimental animals were divided into eight groups according to the random grouping principle: (i) AGS: Lv_pcDNA-NC, Lv_pcDNA-PLLP, Lv_pcDNA-NC + Lv_sh-TP53, and Lv_pcDNA-PLLP + Lv_sh-TP53. (ii) SNU-601: Lv_pcDNA-NC, Lv_pcDNA-PLLP, Lv_pcDNA-NC + Lv_pcDNA-TP53, and Lv_pcDNA-PLLP + Lv_pcDNA-TP53 groups, with seven mice in each group. After lentivirus packaging of the overexpression plasmid or shRNA knockdown sequence (Shanghai GENE), virus infection and stable cell line screening were performed. Afterward, 100 μl of cell suspension (5 × 10^6^ cells) was subcutaneously injected into the left armpit of each mouse to establish tumors. The tumor volume was measured every 3 days, the mice were sacrificed after 18 days, and the tumor tissue weight was measured. The study was performed in accordance with the ethical standards laid out in the 1964 Declaration of Helsinki, and ethics approval was granted from the Experimental Animal Ethics Committee of the Affiliated Hospital of Guangdong Medical University (AHGDMU-LAC-B- 202412-0098).

### Cell proliferation activity analysis

Cell Counting Kit-8 (CCK-8) and colony formation assays were used to analyze cell proliferation. CCK-8: After the cells were treated for 24, 48, or 72 h according to grouping, the supernatant was aspirated and discarded. Afterward, 110 μl/well of diluted CCK-8 working solution (Biosharp) was added, and the cells were incubated at 37 °C and 5% CO_2_ for 2 h. The absorbance values were measured at 450 nm using a microplate reader (ELx800, BioTek). For the colony formation experiment, cell suspensions were seeded at 1000 cells per well in 6-well plates and incubated at 37 °C with 5% CO_2_. The cultures were terminated when macroscopic clones appeared in the 6-well plates. After pure ethanol (Xilong Scientific) was added for fixation, the cells were stained with 0.1% crystal violet (Bomei) for 20 min, images were collected, and the clones were counted.

### Cell invasion analysis

BD Matrigel serum-free medium precooled at 4 °C was diluted at a ratio of 1:8, and 80 μl was removed and added to the upper chamber of the Transwell. After the cells were incubated for 4 to 5 h in the incubator, 100 μl of prewarmed serum-free medium was added to the upper chamber and left for 15 to 30 min. A total of 600 μl of complete medium containing 20% fetal bovine serum was added to the lower chamber, 200 μl of cell suspension (4 × 10^4^ cells/well) was added to the Transwell chamber, and the culture was continued for 24 h. After being fixed with ethanol for 30 min, the cells were stained with 0.1% crystal violet staining solution (Bomei) for 30 min and photographed for counting.

### Apoptosis analysis

Flow cytometry was used to detect apoptosis, and TUNEL staining was used to observe apoptosis in tumor tissues. For flow cytometry, the cell precipitates of each group were obtained, and the cells were resuspended in 500 μl of binding buffer. Then, 5 μl of Annexin V (KeyGEN) was added, and 5 μl of propidium iodide (KeyGEN) was added to the mixture. Reactions were carried out at room temperature in the dark for 15 min and analyzed by flow cytometry (Cytoflex). For TUNEL staining, the tumor tissues of mice were fixed with 4% paraformaldehyde, dehydrated, embedded, sectioned (5 μm), repaired by a citric acid microwave, incubated with fluorescent TUNEL solution (Roche Group) at 37 °C for 1 h, nucleated with 4′,6-diamidino-2-phenylindole for 15 min, and sealed with glycerol. The images of the sections were collected by a microcamera system (DMI1, Leica), and the percentage of positive apoptotic cells in the pictures was calculated.

### Immunohistochemical staining

Immunohistochemical staining was used to observe the expression of PLLP, mutant p53, and WT p53 in clinical tissues and the expression of Ki-67, PLLP, and p53 in tumor tissues. The tissues were fixed with 4% paraformaldehyde, embedded in paraffin, sectioned (5 μm), subjected to antigen repair, blocked with 3% hydrogen peroxide, blocked with bovine serum, and incubated at 4 °C overnight with primary antibodies as follows: PLLP (1:200 dilution, Proteintech); p53-mut (1:50 dilution, Abcam); p53-wt (1:50 dilution, BOSTER); Ki-67 (1:400 dilution, HUABIO); and p53 (1:50 dilution, ABclonal). The sections were then incubated with a secondary antibody (horseradish peroxidase–labeled goat anti-rabbit, Servicebio) at 37 °C for 30 min, followed by 3,3′-diaminobenzidine for color development and hematoxylin counterstaining for 3 min, after which the slides were neutrally gummed. Images were collected by a digital microscope camera system (BA400Digital, Motic), and the proportion of positive area (percent of 3,3′-diaminobenzidine-positive tissue) in each image was calculated by a Halo data analysis system (Halo 101-WL-HALO-1, Indica Labs).

### Quantitative real-time PCR

Samples were collected, total RNA was extracted according to the instructions of the kit (Yeasen), Complementary DNA was synthesized, and qRT–PCR analysis was performed according to the instructions of TB Green Premix Ex Taq II (Tli RNaseH Plus). GAPDH was used as the reference gene. The PCR system consisted of 45 cycles of predenaturation at 95 °C for 30 s, denaturation at 95 °C for 5 s, annealing at 55 °C for 30 s, full extension at 72 °C for 30 s, and fluorescence collection. The primer sequences are shown in Table 3.

**Table 3:** Primer sequences

Gene	Upstream primer (5′-3′)	Downstream primer (5′-3′)
GAPDH	TGACTTCAACAGCGACACCCA	CACCCTGTTGCTGTAGCCAAA
BAX	CGAACTGGACAGTAACATGGAG	CAGTTTGCTGGCAAAGTAGAAA
CDKN1A	GATGGAACTTCGACTTTGTCAC	GTCCACATGGTCTTCCTCTG
PLLP	GCGGCAGTTGACCTGACATC	AGAAGGCACTCACTCCATAGGC
p53	TTCCTGAAAACAACGTTCTGTC	AACCATTGTTCAATATCGTCCG
PUMA	CCTCAACGCACAGTACGA	CCCATGATGAGATTGTACAGGA

### Western blot analysis

Precooled Western and immunoprecipitation (IP) lysis buffer (100 μl; Beyotime) was added to the samples to extract total protein, and the protein concentration was determined by a BCA protein quantification kit (Beyotime). Protein samples were electrophoresed on 12% sodium dodecylsulfate-polyacrylamide gels, transferred to polyvinylidene fluoride membranes, blocked with 5% skim milk for 2 h at room temperature, and incubated with primary antibodies (PLLP [1:1000 dilution, Proteintech]; p53 [1:5000 dilution, Proteintech]; p21 [1:2000 dilution, Proteintech]; BAX [1:2000 dilution, ABclonal]; MDM2 (1:1000 dilution, HUABIO; and TRIM59 [1:1000 dilution, Proteintech]) at 4 °C overnight. The secondary antibody, goat anti-rabbit IgG (H + L) horseradish peroxidase (1:8000 dilution, Affinity), was incubated at room temperature for 2 h and developed by enhanced chemiluminescence, after which the bands were exposed by Tanon automatic chemiluminescence image analysis system software V2.0 (5200 Multi). Gel-Pro Analyzer 4 software (Media Cybernetics, Rockville, MD) was used to scan and express the integrated optical density of the target proteins.

### Coimmunoprecipitation

Coimmunoprecipitation was used to detect the binding between the TRIM59 and PLLP proteins and the ubiquitination of the p53 protein. Cells were harvested and lysed by adding Western and IP cell lysates (Beyotime). The expression of PLLP and p53 in the input protein was detected by immunoblotting (IB), and the ubiquitination level of the p53 protein was subsequently detected after enrichment of the IP:Ub protein with an HA antibody. In addition, IB was used to detect the expression of PLLP, TRIM59, and FLAG-p53 among the input proteins, and after enrichment of the IP:p53 protein with a FLAG antibody, IB was used to detect HA (Ub, HUABIO) to analyze the ubiquitination level of p53. The detection procedure was the same as that for the Western blot analysis.

### Statistical analysis

SPSS 22.0 statistical software was used for statistical analysis. The Shapiro–Wilk normality test was used to test whether the continuous variables conformed to a normal distribution. Data that followed a normal distribution were expressed as the mean ± standard deviation. One-way ANOVA was used to compare the means of multiple samples, the least significant difference test was used for homogeneous variance, and Tamhane's T2 test was used for heterogeneous variance. *p* < 0.05 was considered to indicate a significant difference between groups.

## Data and material availability

The datasets used and analyzed during the current study are available from the corresponding author upon reasonable request. In animal studies, the study was performed in accordance with the ethical standards laid out in the 1964 Declaration of Helsinki, and ethics approval was granted from the Experimental Animal Ethics Committee of the Affiliated Hospital of Guangdong Medical University (AHGDMU-LAC-B-202412-0098).

## Conflict of interest

The authors declare that they have no conflicts of interest with the contents of this article.

## References

[1] López M.J., Carbajal J., Alfaro A.L., Saravia L.G., Zanabria D., Araujo J.M. (2023). Characteristics of gastric cancer around the world. Crit. Rev. Oncol Hematol.

[2] Zhou J., Zheng R., Zhang S., Chen R., Wang S., Sun K. (2022). Gastric and esophageal cancer in China 2000 to 2030: Recent trends and short-term predictions of the future burden. Cancer Med..

[3] Sung H., Ferlay J., Siegel R.L. (2021). Global Cancer Statistics 2020: GLOBOCAN Estimates of Incidence and Mortality Worldwide for 36 Cancers in 185 Countries. CA Cancer J. Clin..

[4] Joshi S.S., Badgwell B.D. (2021). Current treatment and recent progress in gastric cancer. CA Cancer J. Clin..

[5] Qiu H., Cao S., Xu R. (2021). Cancer incidence, mortality, and burden in China: a time-trend analysis and comparison with the United States and United Kingdom based on the global epidemiological data released in 2020. Cancer Commun. (Lond).

[6] Guan W.L., He Y., Xu R.H. (2023). Gastric cancer treatment: recent progress and future perspectives. J. Hematol. Oncol..

[7] Shulgin A.A., Lebedev T.D., Prassolov V.S., Spirin P.V. (2021). Plasmolipin and its role in cell processes. Mol. Biol..

[8] Cacho-Navas C., Reglero-Real N., Colás-Algora N., Barroso S., de Rivas G., Stamatakis K. (2022). Plasmolipin regulates basolateral-to-apical transcytosis of ICAM-1 and leukocyte adhesion in polarized hepatic epithelial cells. Cell Mol. Life Sci..

[9] Kim S.B., Chae G.W., Lee J., Park J., Tak H., Chung J.H. (2007). Activated Notch1 interacts with p53 to inhibit its phosphorylation and transactivation. Cell Death Differ..

[10] Zhou Z., Ji Z., Wang Y., Li J., Cao H., Zhu H.H. (2014). TRIM59 is up-regulated in gastric tumors, promoting ubiquitination and degradation of p53. Gastroenterology.

[11] Kastan M.B. (2007). Wild-type p53: tumors can't stand it. Cell.

[12] Wang S., Zhao Y., Aguilar A., Bernard D., Yang C.Y. (2017). Targeting the MDM2-p53 protein-protein interaction for new cancer therapy: progress and challenges. Cold Spring Harbor Perspect. Med..

[13] Munisamy M., Mukherjee N., Thomas L., Pham A.T., Shakeri A., Zhao Y. (2021). Therapeutic opportunities in cancer therapy: targeting the p53-MDM2/MDMX interactions. Am. J. Cancer Res..

[14] Sun Z., Qiu Z., Wang Z., Chi H., Shan P. (2021). Silencing ribosomal protein L22 promotes proliferation and migration, and inhibits apoptosis of gastric cancer cells by regulating the murine double minute 2-Protein 53 (MDM2-p53) signaling pathway. Med. Sci. Monitor.

[15] Wang H., Guo M., Wei H., Chen Y. (2023). Targeting p53 pathways: mechanisms, structures, and. Adv. Ther..

[16] Muller P.A., Vousden K.H. (2014). Mutant p53 in cancer: new functions and therapeutic opportunities. Cancer Cell.

[17] Donehower L.A., Soussi T., Korkut A., Liu Y., Schultz A., Cardenas M. (2019). Integrated analysis of TP53 gene and pathway alterations in the cancer genome Atlas. Cell Rep..

[18] Olivier M., Hollstein M., Hainaut P. (2010). TP53 mutations in human cancers: origins, consequences, and clinical use. Cold Spring Harbor Perspect. Biol..

[19] Fakhri S., Zachariah Moradi S., DeLiberto L.K., Bishayee A. (2022). Cellular senescence signaling in cancer: a novel therapeutic target to combat human malignancies. Biochem. Pharmacol..

[20] Kaiser A.M., Attardi L.D. (2018). Deconstructing networks of p53-mediated tumor suppression in vivo. Cell Death Differ..

[21] Shangary S., Wang S. (2008). Targeting the MDM2-p53 interaction for cancer therapy. Clin. Cancer Res..

[22] Barajas S., Cai W., Liu Y. (2022). Role of p53 in regulation of hematopoiesis in health and disease. Curr. Opin. Hematol..

[23] Wade M., Li Y.C., Wahl G.M. (2013). MDM2, MDMX and p53 in oncogenesis and cancer therapy. Nat. Rev. Cancer.

[24] Li W.F., Alfason L., Huang C., Tang Y., Qiu L., Miyagishi M. (2023). p52-ZER6: a determinant of tumor cell sensitivity to MDM2-p53 binding inhibitors. Acta Pharmacol. Sin..

[25] Guerreiro N., Jullion A., Ferretti S., Fabre C., Meille C. (2021). Translational Modeling of Anticancer Efficacy to Predict Clinical Outcomes in a First-in-Human Phase 1 Study of MDM2 Inhibitor HDM201. AAPS J..

[26] Vassilev L.T., Vu B.T., Graves B., Carvajal D., Podlaski F., Filipovic Z. (2004). In vivo activation of the p53 pathway by small-molecule antagonists of MDM2. Science (New York, NY).

[27] Pan M., Li X., Tian X., Yang L.Z., Fang W. (2025). Eugenol Alleviates Cerebral Ischemia-Reperfusion Injury in Mice by Promoting the Phagocytosis of Microglia via Up-Regulating Tripartite Motif Protein 59. Basic Clin. Pharmacol. Toxicol..

[28] Wang M., Chao C., Luo G., Wang B., Zhan X., Di D. (2019). Prognostic significance of TRIM59 for cancer patient survival: a systematic review and meta-analysis. Medicine.

[29] Guo J., Min K., Deng L. (2021). Potential value of tripartite motif-containing 59 as a biomarker for predicting the prognosis of patients with lung cancer: A protocol for systematic review and meta-analysis. Medicine (Baltimore).

[30] Yi H., Shi H., Mao W., Yin J., Ma Y., Xu L. (2024). E3 ubiquitin ligase IPI1 controls rice immunity and flowering via both E3 ligase-dependent and -independent pathways. Develop. Cel..

[31] Barretina J., Caponigro G., Stransky N., Venkatesan K., Margolin A.A., Kim S. (2012). The Cancer Cell Line Encyclopedia enables predictive modelling of anticancer drug sensitivity. Nature.

